# Trends and clinical characteristics of 57.9 million COVID-19 vaccine recipients: a federated analysis of patients’ primary care records *in situ* using OpenSAFELY

**DOI:** 10.3399/BJGP.2021.0376

**Published:** 2021-11-09

**Authors:** Helen J Curtis, Peter Inglesby, Caroline E Morton, Brian MacKenna, Amelia Green, William Hulme, Alex J Walker, Jessica Morley, Amir Mehrkar, Seb Bacon, George Hickman, Chris Bates, Richard Croker, David Evans, Tom Ward, Jonathan Cockburn, Simon Davy, Krishnan Bhaskaran, Anna Schultze, Christopher T Rentsch, Elizabeth J Williamson, Anna Rowan, Louis Fisher, Helen I McDonald, Laurie Tomlinson, Rohini Mathur, Henry Drysdale, Rosalind M Eggo, Kevin Wing, Angel YS Wong, Harriet Forbes, John Parry, Frank Hester, Sam Harper, Shaun O’Hanlon, Alex Eavis, Richard Jarvis, Dima Avramov, Paul Griffiths, Aaron Fowles, Nasreen Parkes, Ian J Douglas, Stephen JW Evans, Liam Smeeth, Ben Goldacre, (The OpenSAFELY Collaborative)

**Affiliations:** The DataLab, Nuffield Department of Primary Care Health Sciences, University of Oxford, Oxford.; The DataLab, Nuffield Department of Primary Care Health Sciences, University of Oxford, Oxford.; The DataLab, Nuffield Department of Primary Care Health Sciences, University of Oxford, Oxford.; The DataLab, Nuffield Department of Primary Care Health Sciences, University of Oxford, Oxford.; The DataLab, Nuffield Department of Primary Care Health Sciences, University of Oxford, Oxford.; The DataLab, Nuffield Department of Primary Care Health Sciences, University of Oxford, Oxford.; The DataLab, Nuffield Department of Primary Care Health Sciences, University of Oxford, Oxford.; The DataLab, Nuffield Department of Primary Care Health Sciences, University of Oxford, Oxford.; The DataLab, Nuffield Department of Primary Care Health Sciences, University of Oxford, Oxford.; The DataLab, Nuffield Department of Primary Care Health Sciences, University of Oxford, Oxford.; The DataLab, Nuffield Department of Primary Care Health Sciences, University of Oxford, Oxford.; London School of Hygiene and Tropical Medicine, London.; The DataLab, Nuffield Department of Primary Care Health Sciences, University of Oxford, Oxford.; The DataLab, Nuffield Department of Primary Care Health Sciences, University of Oxford, Oxford.; The DataLab, Nuffield Department of Primary Care Health Sciences, University of Oxford, Oxford.; London School of Hygiene and Tropical Medicine, London.; The DataLab, Nuffield Department of Primary Care Health Sciences, University of Oxford, Oxford.; London School of Hygiene and Tropical Medicine, London.; London School of Hygiene and Tropical Medicine, London.; London School of Hygiene and Tropical Medicine, London.; London School of Hygiene and Tropical Medicine, London.; The DataLab, Nuffield Department of Primary Care Health Sciences, University of Oxford, Oxford.; The DataLab, Nuffield Department of Primary Care Health Sciences, University of Oxford, Oxford.; London School of Hygiene and Tropical Medicine, London.; London School of Hygiene and Tropical Medicine, London.; London School of Hygiene and Tropical Medicine, London.; The DataLab, Nuffield Department of Primary Care Health Sciences, University of Oxford, Oxford.; London School of Hygiene and Tropical Medicine, London.; London School of Hygiene and Tropical Medicine, London.; London School of Hygiene and Tropical Medicine, London.; London School of Hygiene and Tropical Medicine, London.; London School of Hygiene and Tropical Medicine, London.; London School of Hygiene and Tropical Medicine, London.; TPP, Leeds, London School of Hygiene and Tropical Medicine, London.; EMIS Health, Leeds.; EMIS Health, Leeds.; EMIS Health, Leeds.; EMIS Health, Leeds.; EMIS Health, Leeds.; EMIS Health, Leeds.; EMIS Health, Leeds.; London School of Hygiene and Tropical Medicine, London.; London School of Hygiene and Tropical Medicine, London.; London School of Hygiene and Tropical Medicine, London.; The DataLab, Nuffield Department of Primary Care Health Sciences, University of Oxford, Oxford.

**Keywords:** COVID-19, NHS England, SARS-CoV-2, vaccination, general practice, ethnic groups

## Abstract

**Background:**

On 8 December 2020 NHS England administered the first COVID-19 vaccination.

**Aim:**

To describe trends and variation in vaccine coverage in different clinical and demographic groups in the first 100 days of the vaccine rollout.

**Design and setting:**

With the approval of NHS England, a cohort study was conducted of 57.9 million patient records in general practice in England, *in situ* and within the infrastructure of the electronic health record software vendors EMIS and TPP using OpenSAFELY.

**Method:**

Vaccine coverage across various subgroups of Joint Committee on Vaccination and Immunisation (JCVI) priority cohorts is described.

**Results:**

A total of 20 852 692 patients (36.0%) received a vaccine between 8 December 2020 and 17 March 2021. Of patients aged ≥80 years not in a care home (JCVI group 2) 94.7% received a vaccine, but with substantial variation by ethnicity (White 96.2%, Black 68.3%) and deprivation (least deprived 96.6%, most deprived 90.7%). Patients with pre-existing medical conditions were more likely to be vaccinated with two exceptions: severe mental illness (89.5%) and learning disability (91.4%). There were 275 205 vaccine recipients who were identified as care home residents (JCVI group 1; 91.2% coverage). By 17 March, 1 257 914 (6.0%) recipients had a second dose.

**Conclusion:**

The NHS rapidly delivered mass vaccination. In this study a data-monitoring framework was deployed using publicly auditable methods and a secure *in situ* processing model, using linked but pseudonymised patient-level NHS data for 57.9 million patients. Targeted activity may be needed to address lower vaccination coverage observed among certain key groups.

## INTRODUCTION

On 8 December 2020, the NHS in England administered the first COVID-19 vaccination as part of an ambitious vaccine programme to combat the ongoing pandemic owing to severe acute respiratory syndrome coronavirus 2 (SARS-CoV-2). Vaccination is one of the most cost-effective ways of avoiding disease, and worldwide vaccinations prevent 2–3 million deaths per year, but new vaccines can take many years or decades to develop and become part of routine practice.^1^ Since the outset of the COVID-19 pandemic, teams of scientists, clinical trialists, and regulators around the world have worked at unprecedented speed, with >200 vaccines currently being tested.^2^ The UK medicines regulator approved two COVID-19 vaccines for use before the end of 2020: the Pfizer-BioNTech mRNA vaccine and the AstraZeneca-Oxford vaccine.^3^ The Moderna vaccine was subsequently approved in January 2021,^4^ with more vaccine approvals during 2021.

For the UK the independent Joint Committee on Vaccination and Immunisation (JCVI) provides recommendations on vaccinations to the government and England’s NHS. In early December 2020 the JCVI recommended nine priority groups for vaccination (Box 1), largely based on risk of death from COVID-19.^5^ This was the basis for the NHS England vaccination programme, with due recognition that vaccination of care home residents may initially lag behind other groups because of complexities around storing and distributing Pfizer-BioNTech outside of larger healthcare settings without appropriate cold chain facilities.^6^ Vaccinations were administered initially in hospitals and a number of primary care centres; then in GP surgeries, community pharmacies, and newly established mass vaccination centres.

**Box 1.** Priority groups for vaccination advised by the Joint Committee on Vaccination and Immunisation^5^

**Priority group**	**Risk group**
1	Residents in a care home for older adults
	Staff working in care homes for older adults

2	All those aged ≥80 years
	Frontline health and social care workers

3	All those aged ≥75 years

4^a^	All those aged ≥70 years
	Clinically extremely vulnerable individuals (not including pregnant women and those aged <16 years)

5	All those aged ≥65 years

6^b^	Adults aged 16–65 years in an at-risk group

7	All those ≥60 years

8	All those ≥55 years

9	All those ≥50 years

a *Individuals classed as clinically extremely vulnerable (priority group 4) were those advised to ‘shield’ and minimise risk of exposure to infection. They were given a shielding code through national algorithms, their GP, or other healthcare professional.*

b *Those in at-risk groups (priority group 6) were not shielding but had other clinical conditions placing them at increased risk.*

**Table:** How this fits in

The COVID-19 vaccination programme was launched in England on 8 December 2020. Limited information is available on vaccination coverage in detailed demographic and clinical subgroups. This study deployed a data-monitoring framework for vaccine coverage using publicly auditable methods and secure *in situ* processing, for linked but pseudonymised patient-level NHS data on 95% of patients registered with a GP in England. This study highlights lower vaccination coverage in the first 100 days of the vaccine rollout among certain key groups: ethnic minorities, those living in areas of higher deprivation, and individuals living with severe mental illness or learning disabilities where targeted activity may be needed to ensure equitable protection against COVID-19.

Both vaccines administered to date require two doses. Given high infection rates and the relatively high protection thought to be offered by the first dose, after the start of the campaign the JCVI recommended extending the interval to 12 weeks. This strategy was intended to prevent the most deaths and admissions to hospital through maximising the number of patients with some protection against the virus as quickly as possible, although GPs were initially allowed some discretion in the exact timing of the second dose.^7^^,^^8^

OpenSAFELY is a new secure analytics platform for electronic patient records built by the authors’ group on behalf of NHS England to deliver urgent academic and operational research during the pandemic.^9^^,^^10^ Analyses run across all patients’ full-coded pseudonymised primary care records, and includes 57.9 million patients, 95% of people registered with an English general practice, those where EMIS or TPP electronic health record (EHR) software is deployed, with patient-level linkage to various sources, such as secondary care data. Code and analysis are shared openly for inspection and re-use. Vaccine administration details are recorded in the National Immunisation Management Service (NIMS) and electronically transmitted to every individual’s GP record on a daily basis.

OpenSAFELY can provide detailed information about the demographics and clinical conditions of those vaccinated from each patient’s full pseudonymised EHR, which is not available within NIMS. This can reveal whether the vaccine rollout is leaving certain groups behind and whether any targeted action is required to address gaps in coverage.

This study therefore set out to: assess the coverage of COVID-19 vaccination in all patients registered with TPP and EMIS practices in England in near real time in the first 100 days of the campaign; and to describe how coverage varied between key clinical and demographic subgroups.

## METHOD

### Study design

A retrospective cohort study was conducted using general practice primary care EHR data from all England GP practices supplied by the EHR vendors EMIS and TPP. The cohort study began on 8 December 2020, the start of the national vaccination campaign, and ended on 17 March 2021. The authors of the current study are producing weekly vaccine coverage reports with a subset of this data^11^ and will update this analysis regularly with extended follow-up time using near real-time data as the vaccination campaign progresses.

### Data source

Primary care records managed by the GP software providers EMIS and TPP were accessed through OpenSAFELY, an open-source data analytics platform created by the author team on behalf of NHS England to address urgent COVID-19 research questions (https://opensafely.org). OpenSAFELY provides a secure software interface allowing a federated analysis of pseudonymised primary care patient records from England in near real-time within the EMIS and TPP highly secure data environments. Nondisclosive, aggregated results are exported to GitHub where further data processing and analysis takes place. This avoids the need for large volumes of potentially disclosive pseudonymised patient data to be transferred off-site. This, in addition to other technical and organisational controls, minimises any risk of re-identification.

The dataset available to the platform includes pseudonymised data such as coded diagnoses, medications, and physiological parameters. No free-text data are included. All activity on the platform is publicly logged and all analytic code and supporting clinical coding lists are automatically published. In addition, the framework provides assurance that the analysis is reproducible and reusable. Further details on information governance and platform can be found in Supplementary Appendix S1.

### Study population

For the descriptive analysis all patients registered with a general practice using EMIS (*n* = 33 873 987) or TPP (*n* = 24 056 480) in England on 17 March 2021 were included. Patients with unknown date of birth (that is default age >121 years) or unknown sex were excluded.

### Priority groups for vaccination

Patients were classified into their JCVI priority group (Box 1) using SNOMED-CT codelists and logic defined in the national COVID-19 vaccination uptake reporting specification developed by PRIMIS.^12^ Patients were assigned only to their highest priority group and not included again as part of any other priority group. For example, a 76-year-old living in a care home would be assigned to group 1 (care home residents) but not to group 3 (aged 75–79 years). Eligibility was not assessed as defined by occupation, that is, health and care staff for the relevant priority groups (1 and 2) because this information is largely missing from GP records and, where present, is unreliable. These patients were therefore either classified into a lower-priority group where applicable (for example, by clinical conditions or age), or as ‘other’, if a person did not fall into one of the nine defined groups. In line with the national reporting specification, most criteria were ascertained using the latest available data at the time of analysis, with the exception of age, which was calculated as at 31 March 2021 as recommended by Public Health England.^12^

### COVID-19 vaccine status

Vaccination information is transmitted back to patients’ primary care records in the days following vaccine administration in a designated centre. Which patients had any recorded COVID-19 vaccine administration code in their primary care record (only Pfizer-BioNTech mRNA vaccine or AstraZeneca-Oxford vaccine were available at the time of analysis) was ascertained. The latest available date of vaccinations recorded in the most recent comparable OpenSAFELY–EMIS and OpenSAFELY–TPP database build were included for those vaccinated up to 17 March 2021. Any COVID-19 vaccination within 19 days of the first dose was classed as a duplicate record entry, and the first vaccination after this date as the second dose of the schedule.

### Key demographic and clinical characteristics of vaccinated groups

All patient demographics defined by the national reporting specification (for example, ethnicity) were extracted. Demographics not defined by the specification, including the level of deprivation, were also extracted. Deprivation was measured by the Index of Multiple Deprivation (IMD, in quintiles, with higher values indicating greater deprivation), derived from the patient’s postcode at Lower Super Output Area. Patients with missing data were grouped into an unknown category.

The population was also described according to the presence or absence of various pre-existing health problems: chronic cardiac disease; diabetes; chronic kidney disease; severe mental illness; learning disabilities; chronic neurological disease (including stroke); asplenia; morbid obesity; chronic liver disease; chronic respiratory disease; and immunosuppression. Patients lacking codes in their primary care record indicating these conditions were assumed to be free of these conditions.

### Factors associated with time to COVID-19 vaccination

To examine relationships between multiple patient characteristics in older adults and the chance of receiving a first COVID-19 vaccine by 17 March 2021, a federated survival analysis was carried out with individual Cox regression models fitted to the TPP and EMIS datasets, before combining model coefficients using inverse-variance-weighting. Here, patients were included who were registered in England and aged ≥70 years on 7 December 2020 (*n* = 7 152 830, Supplementary Figure S1) and censored at death or deregistration. Patients aged <70 years were excluded because of the inability to ascertain health/care worker status, a strong determinant of early vaccination. Patients with <1 year of prior follow-up were excluded to ensure completeness of clinical records (*n* = 237 940), and care home or nursing home residents (*n* = 154 123) were excluded as the vaccine rollout was organised separately for these individuals. All key demographic and clinical characteristics described in the section above were included in the multivariable model, with age grouped into 5-year age bands. Patients with missing sex or IMD (*n* = 59 386) were excluded. The model was stratified by GP practice. Hazard ratios from the fully adjusted model are reported with 95% confidence intervals (CIs).

### Codelists and implementation

Information on all characteristics were obtained from primary care records by searching TPP SystmOne and EMIS records for specific coded data. EMIS and TPP SystmOne are fully compliant with the mandated NHS standard of SNOMED-CT clinical terminology. Medicines are entered or prescribed in a format compliant with the NHS Dictionary of Medicines and Devices (dm+d).^13^ Codelists and logic for most features in the national reporting specification were automatically converted to software (https://codelists.opensafely.org/codelist/primis-covid19-vacc-uptake).

### Analysis

Charts of vaccine coverage were generated for all underlying conditions and medicines. Those not presented in this manuscript are available online for inspection in the associated GitHub repository.^14^

### Software and reproducibility

Data management and analysis was performed using the OpenSAFELY software libraries and Jupyter notebooks, implemented using Python 3 and R (version 4.0.2). More details are available in Supplementary Data and openly accessible GitHub repository. This is the first analysis delivered using federated analysis through the OpenSAFELY platform: codelists and code for data management and data analysis were specified once using the OpenSAFELY tools; then transmitted securely from the OpenSAFELY jobs server to the OpenSAFELY–TPP platform within TPP’s secure environment, and separately to the OpenSAFELY–EMIS platform within EMIS’s secure environment, where they were each executed separately against local patient data; summary results were then reviewed for disclosiveness, released, and combined for the final outputs.

All code for the OpenSAFELY platform for data management, analysis, and secure code execution is shared for review and re-use under open licences at GitHub.com/OpenSAFELY. All code for data management and analysis for this article is shared for scientific review and re-use under open licences on GitHub (https://github.com/opensafely/covid19-vaccine-coverage-tppemis).

### Patient and public involvement

Patients were not formally involved in developing this specific study design that was developed rapidly in the context of the rapid vaccine rollout during a global health emergency. The authors have developed a publicly available website (https://opensafely.org) through which any patient or member of the public is invited to make contact regarding this study or the broader OpenSAFELY project.

## RESULTS

### COVID-19 vaccine coverage in JCVI priority groups

**Figure 1.**
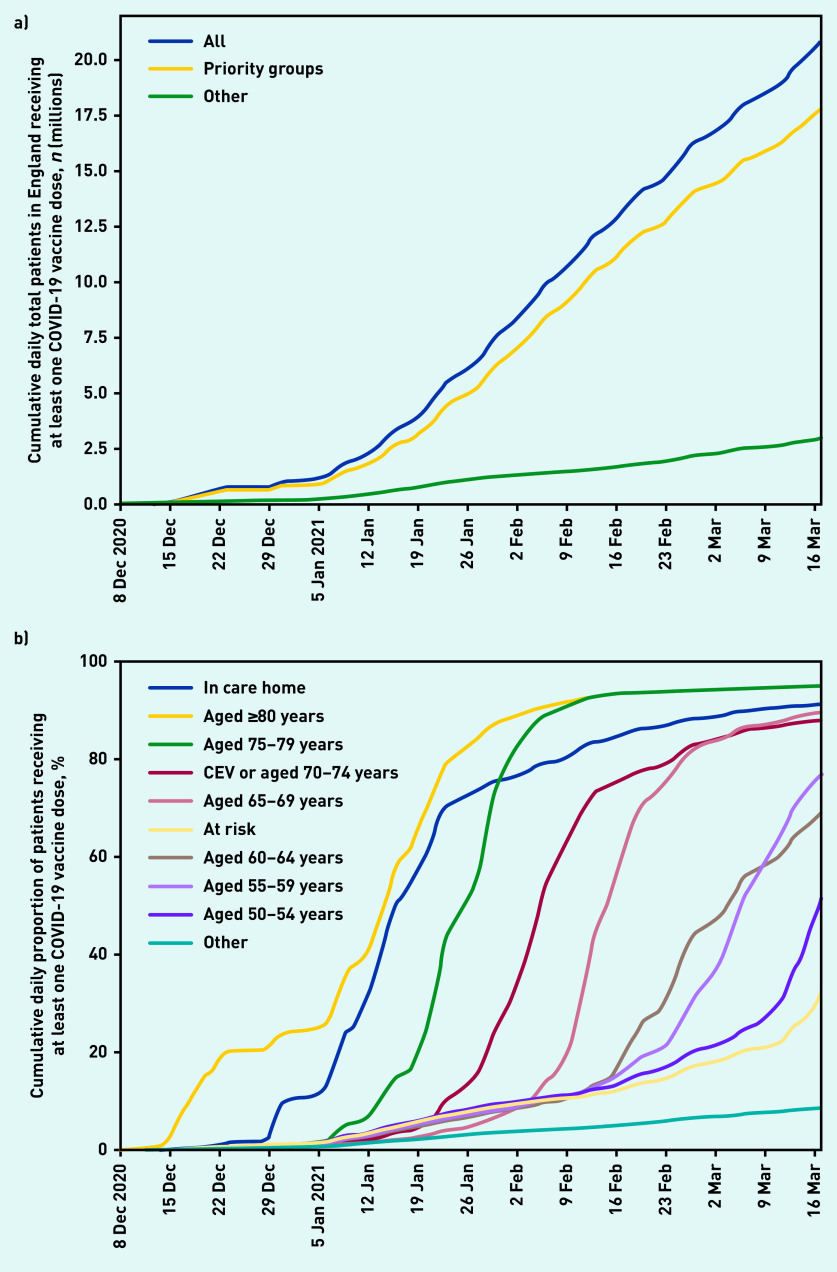
*a) Cumulative daily total number of patients (millions) in England receiving at least one vaccination recorded in OpenSAFELY on 17 March 2021; across the whole population (All) and split according to whether patients were known to be part of a priority group or not (excluding occupational eligibility). b) Cumulative daily proportion (%) of patients receiving at least one COVID vaccine dose by Joint Committee on Vaccination and Immunisation priority groups (excluding occupational eligibility) in England recorded in OpenSAFELY on 17 March 2021.* *CEV = clinically extremely vulnerable.*

A total of 20 852 692 (36.0%) patients registered at an EMIS or TPP practice received their first COVID-19 vaccine up to 17 March 2021 (Table 1, Figure 1). Of these, 275 205 were identified as living in a care home (group 1, 91.2% coverage of all care home residents), 2 422 476 were aged ≥80 years (group 2, 94.7% of all those aged ≥80 years not identified as living in a care home); and 1 865 927 were aged 75–79 years (group 3, 94.7% coverage).

**Table 1.** Vaccination coverage among 95% of the population of registered patients in England, according to COVID-19 vaccination priority groups, at 17 March 2021^a^

**Priority group**	**Vaccinated at 17 March, %**	**Vaccinated at 17 March, *n***	**Population**	**Vaccinated at 10 March, %**	**Increase in coverage in latest week, % point**
1. In care home	91.2	275 205	301 889	90.4	0.7
2. Aged ≥80 years	94.7	2 422 476	2 558 906	94.5	0.2
3. Aged 75–79 years	94.7	1 865 927	1 970 234	94.5	0.2
4. CEV or aged 70–74 years	87.7	4 113 627	4 690 203	86.7	1.0
5. Aged 65–69 years	89.4	2 210 110	2 472 862	87.6	1.7
6. At-risk	68.7	2 861 474	4 165 987	60.1	8.6
7. Aged 60–64 years	76.9	1 644 636	2 139 200	63.7	13.2
8. Aged 55–59 years	51.6	1 438 913	2 790 228	30.2	21.4
9. Aged 50–54 years	32.5	1 008 273	3 097 668	22.1	10.5
Other	8.9	3 012 051	33 743 290	8.0	1.0
Population	36.0	20 852 692	57 930 467	32.6	3.4

a *All ages are included. CEV = clinically extremely vulnerable.*

In later priority groups, 4 113 627 were identified as aged 70–74 years or clinically extremely vulnerable (group 4, 87.7% coverage); 2 210 110 aged 65–69 years (group 5, 89.4% coverage); and all other priority groups had at least 32.5% vaccination coverage. Among the youngest of the priority groups (50–54 and 55–59 years of age) some of those vaccinated to date are likely health and care workers. A total of 3 012 051 patients, most likely health and care workers, were identified as being vaccinated but not assigned to one of the priority groups. Of all those vaccinated, 12 080 194 (57.9%) received the AstraZeneca-Oxford vaccine as their first dose, 8 691 536 (41.7%) the Pfizer-BioNTech vaccine, with the remainder receiving a vaccine where the brand was not specified (*n* = 80 962). There were 1 257 914 patients who received a second dose (6.0%) (data available from the GitHub repository).^14^

### Key demographic and clinical characteristics of priority group 2

**Figure 2.**
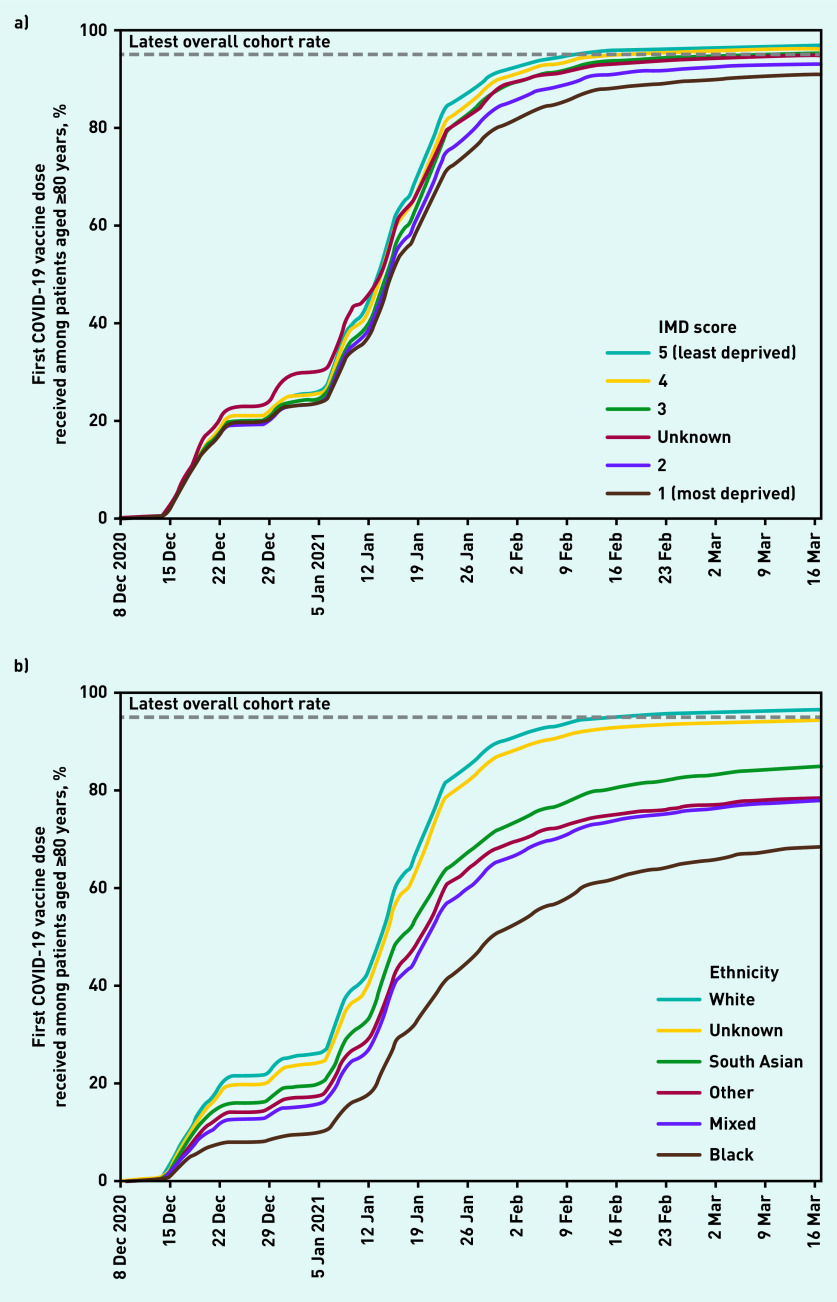
*Cumulative trends in demographic features of the 80 years and older population receiving their first COVID vaccination. a) COVID vaccinations among the age 80 years and over population by Index of Multiple Deprivation. b) COVID vaccinations among the age 80 years and over population by ethnicity (broad categories).* *IMD = Index of Multiple Deprivation.*

**Figure 3.**
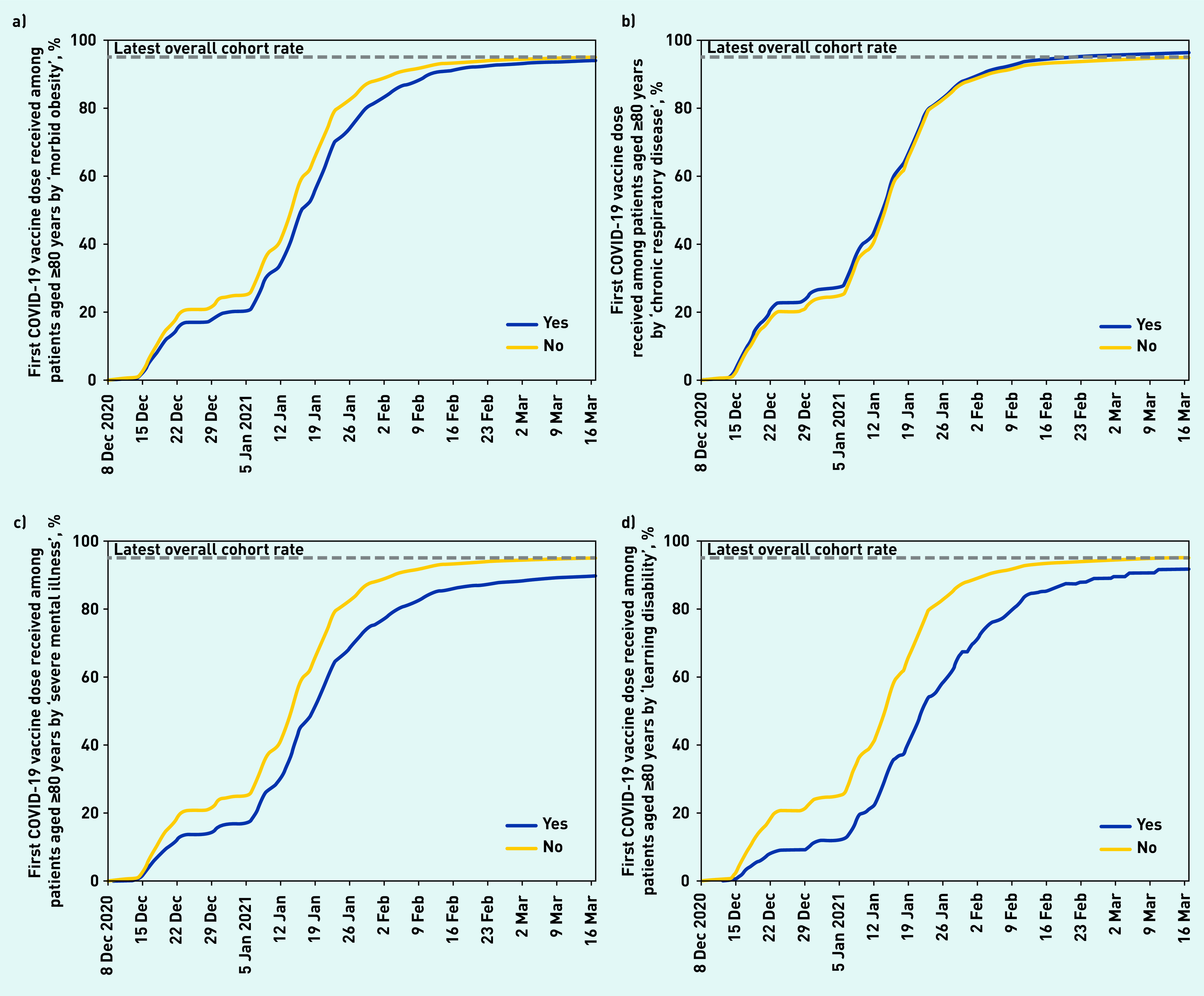
*Cumulative trends in clinical characteristics of priority group 2 (age 80 years and over population and not resident in a care home) receiving their first COVID vaccination. a) COVID vaccinations among the age 80 years and over population by ‘morbid obesity’. b) COVID vaccinations among the age 80 years and over population by ‘chronic respiratory disease’. c) COVID vaccinations among the age 80 years and over population by ‘severe mental illness’. d) COVID vaccinations among the age 80 years and over population by ‘learning disability’.*

The substantially larger priority group 2 was offered vaccination alongside group 1. Priority group 2 included those aged ≥80 years who were not known to be living in a care home, and rapidly reached 90% coverage over the first 8 weeks of the vaccination campaign, with a further increase of 4.7% over the remaining weeks of the study period (data available from the GitHub repository).^14^ A breakdown of the proportion of patients vaccinated by 17 March in this group by various demographic and clinical categories is provided in Table 2 and Figures 2 and 3. Vaccination was less common among those living in the most deprived postcode areas (90.7% in the most deprived quintile compared to 96.6% in the least deprived, Figure 2a).

**Table 2.** COVID vaccinations among priority group 2 (aged 80 years and over population not resident in care homes) as at 17 March 2021, according to demographic and clinical features^a^

	**Vaccination, % at 17 March**	**Vaccination, *n* at 17 March**	**Population, *n***	**Vaccination, % 10 March**	**Increase in coverage in latest week, %**
**Overall**	94.7	2 422 476	2 558 906	94.5	0.2

**Sex**					
Female	94.5	1 400 532	1 481 970	94.3	0.2
Male	94.9	1 021 944	1 076 936	94.8	0.1

**Ethnicity**					
White — British	96.7	1 391 404	1 439 613	96.5	0.1
White — Irish	93.6	19 579	20 923	93.4	0.2
White — any other White background	86.5	47 551	54 992	86.3	0.2
Mixed — White and Black Caribbean	75.0	2779	3703	74.5	0.6
Mixed — White and Black African	68.3	798	1169	67.1	1.2
Mixed — White and Asian	86.2	1134	1316	85.6	0.5
Mixed — any other mixed background	81.5	1918	2352	81.2	0.3
Asian or Asian British — Indian	89.9	29 456	32 753	89.7	0.2
Asian or Asian British — Pakistani	76.9	12 859	16 723	76.0	0.9
Asian or Asian British — Bangladeshi	81.3	3969	4879	80.5	0.9
Asian or Asian British — any other Asian background	82.3	9548	11 599	82.0	0.4
Black or Black British — Caribbean	71.3	16 681	23 387	70.6	0.7
Black or Black British — African	59.8	5152	8617	59.0	0.8
Black or Black British — any other Black background	69.8	1750	2506	69.0	0.8
Other ethnic groups — Chinese	78.1	3199	4095	77.9	0.2
Other ethnic groups — any other ethnic group	78.3	5348	6832	77.8	0.5
Patients with any other ethnicity code	95.4	377 566	395 654	95.3	0.2
Ethnicity not stated	92.9	15 344	16 513	92.7	0.2
Ethnicity not recorded	93.2	476 336	511 182	93.0	0.2

**Ethnicity (broad categories)**					
White	96.2	1 458 548	1 515 535	96.1	0.1
Mixed	77.7	6650	8554	77.3	0.5
South Asian	84.6	55 846	65 975	84.2	0.5
Black	68.3	23 590	34 517	67.6	0.8
Other	78.3	8561	10 934	77.9	0.4
Unknown	94.1	869 260	923 363	94.0	0.2

**Index of Multiple Deprivation**					
1 (most deprived)	90.7	309 190	340 928	90.4	0.3
2	92.9	402 836	433 622	92.7	0.2
3	95.0	514 682	541 737	94.8	0.2
4	95.9	564 410	588 546	95.8	0.1
5 (least deprived)	96.6	612 731	634 340	96.5	0.1
Unknown	94.5	18 613	19 691	94.3	0.2

**Clinical risk groups**					
Patients in any clinical risk group	95.7	1 766 590	1 846 677	95.5	0.2
Patients with immunosuppression	96.6	107 184	110 999	96.4	0.2
Patients with CKD	95.9	750 820	782 705	95.8	0.2
Patients who have chronic respiratory disease	96.0	294 413	306 810	95.8	0.2
Patients with diabetes	94.8	492 982	519 862	94.6	0.2
Patients with chronic liver disease	95.4	44 618	46 746	95.2	0.2
Patients with chronic neurological disease (including stroke/TIA)	95.4	408 730	428 400	95.2	0.2
Patients with chronic heart disease	96.1	1 042 034	1 084 643	95.9	0.2
Patients with asplenia or dysfunction of the spleen	96.8	19 950	20 615	96.7	0.1
Patients with learning disability	91.4	1190	1302	91.4	0.0
Patients with severe mental health	89.5	15 582	17 409	89.1	0.4

**Other groups**					
Patients who are clinically extremely vulnerable	93.6	706 629	754 789	93.4	0.2
Patients with morbid obesity	93.8	32 837	35 014	93.5	0.3

a *Values <7 suppressed. Patient counts are rounded to the nearest 7. CKD = chronic kidney disease. TIA = transient ischaemic attack.*

Across broad ethnic groups (Figure 2b), the proportion vaccinated to date was highest among White ethnicity (96.2%); lowest among Black ethnicity (68.3%); 77.7%–84.6% among patients of mixed, other, and South Asian ethnicities; and 94.1% among those with unknown ethnicity; ethnicity coding was 63.9% complete in this age group. Vaccination coverage in detailed ethnic groups ranges from 96.7% in the White — British group to 59.8% in the Black or Black British — African group (Table 2).

Vaccination coverage was initially lower among those with obesity (Figure 3a); however, this gap had largely resolved by mid-March 2021. Vaccination coverage was slightly higher among patients with other physical comorbidities such as chronic respiratory disease (Figure 3b), cardiac disease, or chronic kidney disease. Vaccination coverage was substantially lower among those living with severe mental illness (89.5%, Figure 3c) and learning disabilities (91.4%, Figure 3d), with some improvements over time.

### Key demographic and clinical characteristics of other priority groups

Detailed tables and charts of breakdowns for all other priority groups are available on the GitHub repository.^14^ The differences by demographic and clinical features observed in priority group 2 were broadly reflected in other priority groups. Key exceptions were that the lower-priority groups, not yet widely vaccinated, had a different pattern of discrepancy by ethnicity, with the South Asian population most vaccinated. This pattern was also seen in several other groups during early stages of the campaign, before widespread targeting of individual groups, such as the 70–74 and 75–79 age groups. Recent improvements in the vaccination coverage of the Bangladeshi community was also noted, where an acceleration with respect to other ethnic groups was seen from around 16 February, particularly noticeable in priority groups 4, 6, 7, 8, and 9.

### Factors associated with time to COVID-19 vaccination

**Figure 4.**
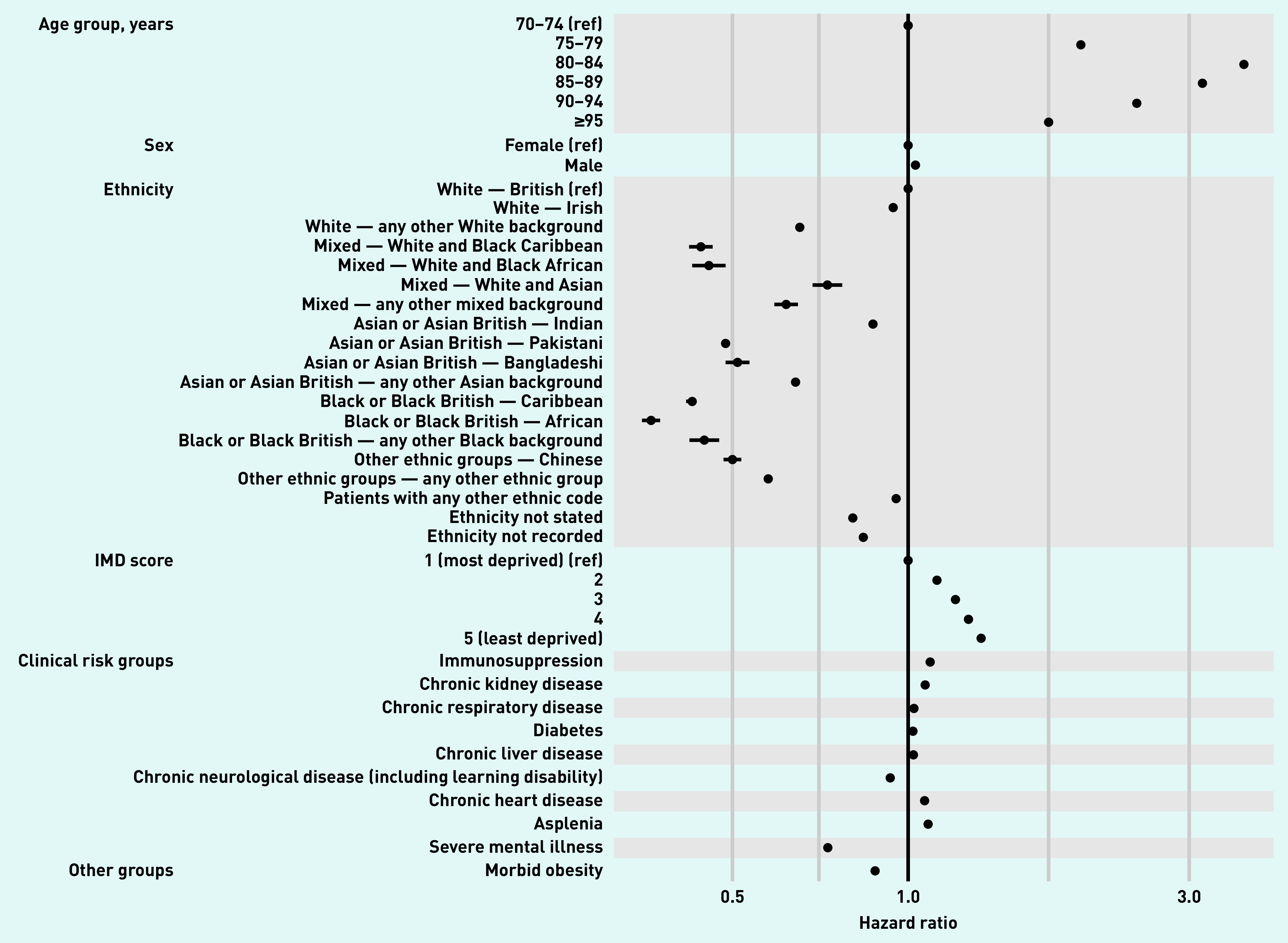
*Estimated hazard ratios (HR) for each potential risk factor from a multivariable Cox model, stratified by GP practice. All HRs are adjusted for all other factors listed.* *IMD = Index of Multiple Deprivation.*

For the multivariable model, 6 701 393 people aged ≥70 years were included (Supplementary Figure S1). Associations between patient-level factors and having a COVID-19 vaccine are shown in Figure 4 and Supplementary Table S1. Of note, after adjustment for modelled demographic and clinical characteristics, the chance of being vaccinated was significantly higher in those living in less deprived areas (least deprived IMD quintile versus most deprived hazard ratio [HR] 1.31, 95% CI = 1.31 to 1.32; *P*<0.0001), and smaller in those from ethnic minority groups (for example, compared with White — any other White background, HR 0.69, 95% CI = 0.69 to 0.70; Black or Black British — African, HR 0.40, 95% CI = 0.40 to 0.41).

All of the clinical risk groups were associated with a higher chance of being vaccinated, with the exception of chronic neurological diseases (HR 0.93, 95% CI = 0.93 to 0.93) and severe mental illness (HR 0.75, 95% CI = 0.74 to 0.75) (Figure 4).

## DISCUSSION

### Summary

The NHS in England has rapidly responded to the availability of COVID-19 vaccines and administered a substantial number of doses in the first 100 days of the vaccination campaign. In this study, 20 852 692 patients (36.0% of patients registered in 97% of GP practices in England) received at least one vaccine dose by 17 March 2021, including 94.7% of eligible patients aged ≥80 years (priority group 2). However, ethnic minorities in priority group 2 were substantially less likely to be vaccinated, and those living in more socioeconomically deprived areas generally had lower vaccine coverage. Similarly, these patterns were broadly observed across the majority of priority groups. Furthermore, in those aged ≥80 years, patients with pre-existing medical conditions were equally likely, or more likely, to have received a vaccine, across all groups of pre-existing medical problems, with two exceptions: vaccination was lower among patients living with severe mental illness and learning disabilities. Similarly, in a risk-adjusted model, chance of vaccination was significantly lower among those living in deprived areas, those from ethnic minority groups, those with chronic neurological disease (including learning disability), and those with severe mental illness.

A total of 3 012 051 people who received a vaccination, most likely health and care workers, were not identified as part of any priority group. Usage was split between two vaccine brands, Pfizer-BioNTech (41.7%, *n* = 8 691 536) and AstraZeneca-Oxford (57.9%, *n* = 12 080 194). A second dose of the vaccine was received by 6.0% (*n* = 1 257 914).

### Strengths and limitations

The key strengths of this study are the scale, detail, completeness, and timeliness of the underlying raw EHR data. This analysis was executed across the full dataset of all raw, pseudonymised, single-event-level clinical events for 57.9 million patients registered at all NHS GP practices in England using EMIS and TPP software; this includes data on all tests, treatments, diagnostic events, and other salient clinical and demographic information. This was achieved by developing and deploying data management and data analysis software inside the EHR vendors’ infrastructure, where the patient data already resides. As a consequence of this, OpenSAFELY can deliver insights into health service activity and clinical outcomes in near real time: raw data can be processed into a completed analysis within days of a clinical event being entered into the patient’s record.

Another key strength is that all eligible patients were identified in each JCVI priority group by directly implementing the full official SNOMED-CT codelists and logic for the national PRIMIS COVID-19 vaccination uptake reporting specification, thus ensuring that the cohorts are perfectly in line with national procedures and GP expectations.

Some limitations to this analysis are recognised. The population, although extremely large, may not be fully representative of the full eligible population: it does not include individuals not registered with a general practice; or the 4% of patients registered at practices not using TPP and EMIS. Primary care records, while detailed and longitudinal, can be incomplete on certain patient characteristics.

Occupation is generally not available in the EHR so it was not possible to assess the eligibility in priority groups 1 or 2 where this is based on occupation. This means that it was not possible to determine the appropriateness of vaccination for those in the ‘other’ group. For patients aged ≥80 years, 36.2% and 0.8% had missing ethnicity or IMD information, respectively. In the weekly COVID-19 vaccination coverage report a different ethnicity codelist and additional sources of data within OpenSAFELY-TPP are used to reduce missing data to <10% for ethnicity.^11^ This method will be implemented within OpenSAFELY-EMIS when possible.

As a federated analysis has been carried out across two EHR vendor’s systems, it is possible that a very small number of patient records are duplicated; however, this has now been established to represent <0.03% of the total patient count. The ascertainment of vaccination status relies on the vaccination administration electronic message being successfully received into the primary care record; while these numbers are consistent with national figures, methods are being explored to also cross-validate this against other sources of person-level vaccination data, broken down by vaccination site type.

Finally, there is currently no well-validated person-level data to identify individuals resident in a care home: this is a limitation for all UK healthcare database studies. The method used for identifying care home residents in this analysis — a clinical code as detailed by the national reporting specification — will lead to under-ascertainment.^15^ The authors of the current study are launching a programme of work, in collaboration with the UK health data science community, to describe and validate the best methods for identifying current care home residents, to produce a better understanding of their health outcomes.

### Comparison with existing literature

The UK had already administered 40.49 vaccines per 100 people by 17 March 2021, one of the fastest vaccination programmes in the world.^16^ NHS England publish report counts of vaccines delivered using vaccination data extracted from the NIMS and report that 21 886 125 first dose vaccinations had been administered by 17 March 2021:^17^ this is in line with the finding in this current study of 20 852 692 patients vaccinated using OpenSAFELY data. It is reasonable to expect marginal differences in the count of patients vaccinated, and the proportions vaccinated, between different summary reports from different analytic teams, because of minor differences in the speeds of data flow, and in the ascertainment of denominators. The total OpenSAFELY population figure is 57.9 million, which is approximately 95% of the NHS Digital estimate for the number of people registered in England with an NHS GP in March 2021 (60.65 million).^18^ It is noted that this figure is higher than the latest Office for National Statistics (ONS) estimated population of England (56.2 million):^19^ the difference between ONS population estimates and NHS-registered populations is a well-recognised issue and may be caused by over-counting at GP practices, differences in definition, and under-counting by the ONS, or a combination of all three.^20^^,^^21^

To the authors’ knowledge, this manuscript, an update of the 27 January 2021 preprint covering only OpenSAFELY-TPP patients (40% of the population),^22^ is the first study to describe in detail the demographic and clinical features of those who have been vaccinated by the NHS England COVID-19 vaccination campaign; and the only study to report on variation in vaccination by fine-grained clinical characteristics, because OpenSAFELY can provide detailed information about the demographics and clinical conditions of those vaccinated from each patient’s full pseudonymised EHR, which is not available within NIMS.

The finding of lower vaccination coverage in Black and Asian groups is concerning: it is also consistent with previous research on variation in vaccine coverage between ethnic groups during other vaccination campaigns historically,^23^^–^25 and with survey data on intention to accept the COVID-19 vaccine.^26^^–^29 The association reported here with deprivation (aged ≥80 years: most deprived 90.7%, least deprived 96.6% [5.9% difference]) resembles other national vaccination campaigns (for example, pneumococcal polysaccharide vaccine) of coverage in those aged ≥65 years: most deprived 68.4%, least deprived 70.9% [2.5% difference]; shingles coverage at age 70 years: 41.0% versus 46.4% [5.4% difference]),^30^ and may somewhat even out during the course of the rapid vaccine rollout.

### Implications for research and practice

The reasons underpinning variation in COVID-19 vaccination coverage are not yet understood, and information presented here should not be misinterpreted as a criticism of the rapidly established NHS vaccination campaign. Further research is needed to understand and address the observed lower vaccination coverage among patients from more deprived areas, and the striking disparity between ethnic groups. The initial preprint on 27 January 2021 and this author group’s regular updates^11^^,^^22^ have received substantial media coverage, particularly with regards to the differences in vaccinations between different ethnic communities.^31^^–^34 The NHS, government, and communities themselves have introduced targeted activities to address the gap including vaccination at places of worship,^35^ webinars led by community leaders to tackle misinformation,^36^ and targeted funding for groups with remit for tackling any health inequalities.^37^^,^^38^ The authors note the accelerated increase in the Bangladeshi community from mid-February, which may represent targeted action by a community group and/or a local NHS organisation. The regular OpenSAFELY vaccination coverage reports can support assessment of the success of these activities in increasing vaccination coverage.

It is reassuring to see that those with a previous history of various medical problems are being vaccinated at the same rate as other patients: in particular it is reassuring to see no evidence that the vaccine programme is currently missing those with serious physical health problems who are at highest risk of death from COVID-19. The lower vaccination coverage among patients living with severe mental illness and learning disabilities is concerning: this may reflect challenges around access, including for those currently living in institutional settings. However, in the latest weeks there was evidence of the vaccination gap narrowing in these groups (Figures 3c and 3d). In late February the JCVI recommended expanded vaccine access for all people on the GP learning disability register as well as adults with other related conditions, including cerebral palsy. This update of the previous advice was based on OpenSAFELY analysis showing a higher risk of mortality in those with learning disabilities^39^ and the JCVI anticipated that an additional 150 000 people would receive the vaccine sooner as a result of this advice.^40^

The authors note that all findings in the present study are from the first 100 days of a major national vaccination programme. Very substantial changes in coverage among different groups are to be expected over the coming months. Weekly vaccine coverage reports are being shared by this author group to assist in monitoring and targeting vaccine initiatives along with machine-readable outputs for re-use in different formats (www.opensafely.org/covid-vaccine-coverage).

More broadly, the UK has an unusually large volume of very detailed longitudinal patient data, especially through primary care. The authors believe the UK has a responsibility to the global community to ensure that these data are used to inform response to the COVID-19 pandemic, in a timely manner, while maintaining the security of individual health records and ensuring the full transparency of all actions on the data to build public trust. To this end, codelists and code for data management and data analysis can only be executed on OpenSAFELY after first being made available at GitHub.com/openSAFELY before execution; this is then shared publicly under open licences for review and re-use either at, or before, the time when results are reported.

In conclusion, the NHS in England has rapidly deployed a mass vaccination campaign. Targeted activity may be needed to address lower vaccination coverage observed among certain key groups: ethnic minorities, those living in areas of higher deprivation, and individuals living with severe mental illness or learning disabilities. Live data monitoring is likely to help support those on the frontline making complex operational decisions around vaccine rollout.
